# Feasibility of severe acute respiratory syndrome coronavirus 2 (SARS-CoV-2) antigen self-testing in school and summer camp attendees

**DOI:** 10.3389/fped.2022.975454

**Published:** 2023-01-10

**Authors:** Andreu Colom-Cadena, Héctor Martínez-Riveros, Anna Bordas, Lucia Alonso-García, Marcos Montoro-Fernández, Pol Romano-deGea, Josep Vidal-Alaball, Elisabet Solà-Segura, Josep M. Llibre, Boris Revollo, Jordi Casabona, Cristina Agustí

**Affiliations:** ^1^ Centre of Epidemiological Studies on Sexually Transmitted Infections and AIDS of Catalonia (CEEISCAT), Ministry of Health, Government of Catalonia, Badalona, Spain; ^2^ Institut d’Investigació Germans Trias I Pujol (IGTP), Badalona, Spain; ^3^ Doctorate Program in Methodology of Biomedical Research and Public Health, Department of Pediatrics, Obstetrics and Gynecology and Preventive Medicine, Univ Autonòma de Barcelona, Badalona, Spain; ^4^ Health Promotion in Rural Areas Research Group, Gerència Territorial de la Catalunya Central, Institut Català de la Salut, Sant Fruitós del Bages, Spain; ^5^ Unitat de Suport a la Recerca de la Catalunya Central, Fundació Institut Universitari per a la Recerca a l'Atenció Primària de Salut Jordi Gol I Gurina, Sant Fruitós del Bages, Spain; ^6^ University of Vic-Central University of Catalonia, Vic, Spain; ^7^ Primary Heatlhcare Team Vic Nord, Institut Català de la Salut, Vic, Spain; ^8^ Division of Infectious Diseases and Foundation for Fighting AIDS, Infectious Diseases and Promoting Health and Science, University Hospital Germans Trias I Pujol, Badalona, Spain; ^9^ Departament de Pediatria, d’Obstetrícia I Ginecologia I de Medicina Preventiva I de Salut Publica, Universitat Autònoma de Barcelona, Bellaterra, Spain; ^10^ Spanish Consortium for Research on Epidemiology and Public Health (CIBERESP), Instituto de Salud Carlos III, Madrid, Spain

**Keywords:** acceptability and usability, SARS-CoV-2 antigen testing, SARS-CoV-2, school public health, self-testing

## Abstract

**Background:**

SARS-CoV-2 screening is one of the pillars of non-pharmaceutical preventive strategies to early identify and isolate infected individuals and therefore decrease community incidence.

**Methods:**

We assessed the feasibility of severe acute respiratory syndrome coronavirus 2 self-testing with antigen-detecting rapid diagnostic tests in attendees of educational settings.

**Results:**

A total of 305 students (88.15%) and 41 staff (11.85%) from 9 to 56 years old participated in the self-testing procedure and answered the survey at the end of the study. 91.3% (*n* = 313) did not need help, 96.1% of participants reported the same outcome as the healthcare workers. 94.5% strongly or slightly agree with the statement “I would repeat the experience”.

**Conclusion:**

The study demonstrates that self-testing is acceptable and usable in children, adolescents and adults when the epidemiological situation may require a systematic screening of these populations, although supervision by health care or previously trained personnel is recommended for younger age groups.

## Introduction

Since the emergence of SARS-CoV-2 in December 2019 governments have implemented various measures to control the spread of the virus. Schools were severely affected and initially closed in many countries, including Spain, despite uncertainty if school closures were an effective containment measure, with a negative impact on the education of children and adolescents (1).

However, data from different countries showed that reopening or never closing schools was not necessarily associated with a significant increase in child-to-child or community transmission in children under 14 years of age (2).

Measures must be adapted to each setting to prevent transmission of the virus (3). An important strategy to minimize SARS-CoV-2 transmission is the rapid identification of infected people, symptomatic or not. Non-pharmaceutical preventive interventions such as screening asymptomatic people for SARS-CoV-2, have been shown to decrease incidence at the community level (3). Antigen detection rapid diagnostic tests (Ag-RDTs) have been proposed as suitable for point-of-care screening of potentially exposed people. Advantages of Ag-RDTs include: low price, absence of referral to a high-tech laboratory, short turnaround time for results and identification of people with potential to transmit their SARS-CoV-2 infection (3) However, taking into account that rapid tests are often developed and marketed when outbreaks are already advanced, should be considered as a medium to long term measure. Making Ag-RDTs available in educational centers could reduce care school closures, costs, response time and eventually SARS-CoV-2 transmission.

As nasal Ag-RDT self-testing is considered to be reliable and feasible in adults (4, 5), the main objective of this study was to assess whether it is acceptable and usable in younger ages, by focusing on students and staff in schools and summer camps during the fifth wave caused by the Delta B.1.617.2 variant of SARS-CoV-2 in Catalonia (Spain).

## Materials and methods

### Study population and period

The research took place between April and August 2021 in 2 schools [one from COVID-19 Sentinel Schools Network of Catalonia (6) and other school from ESCORAT project] and 4 summer camps in Catalonia, Spain. Thus, here students over 9 years old were invited to participate and staff were included only in summer camps. In one school (School A) a prospective cohort was established (testing weekly for 8 weeks) while a cross-sectional study was done in School B and summer camps. The field team consisted of between 2 and 4 healthcare workers (nurses and nursing assistants).

### Material delivery and data collection

After signing the informed consent, participants received a sampling kit including: paper-based schematic and illustrated instructions for the self-testing procedure, a printed feasibility survey, ID labels and the SARS-CoV-2 Ag-RDT Kit [test cassette, nasal swab, empty tube and plug, Pasteur pipette and buffer solution; Panbio™ COVID-19 Ag Rapid Test Device (Abbot Laboratories, Chicago, US)]. Additionally, the self-test procedure was recorded on video and distributed to participating schools.

Tests were performed in accordance with biosecurity measures (well-ventilated area, separation between participants of >1.5 m, table disinfection with alcohol before and after the procedures). Subsequently, acceptability surveys were entered into the EUSurvey platform by the research team.

All test results were validated. In School B and summer camps, participants read the test result themselves, and the healthcare team validated the reliability, while in School A previously trained older pupils (15–16 years old) supervised the sampling procedure and read the results.

Ag-RDT Positive cases were referred to the health center for a Reverse Transcription-PCR (RT-PCR) with nasal swab to confirm the results.

### Feasibility evaluation

We assessed the acceptability and usability of the intervention among participants based on a conceptual validated framework adapted from previous studies (7). Here, acceptance and use of SARS-CoV-2 Ag-RDT self-testing was adapted and divided into the following subdomains: *Learnability, Willingness, Suitability, Satisfaction and Efficacy* (see Table 1).

**Table 1:** Acceptability and usability subdomains results by age groups. *N* = 346, April-August 2021, Catalonia (Spain).

				Participants by age range				
			Total *N* (%)	9–11 years old *N* (%)	12–15 years old *N* (%)	16–18 years old *N* (%)	19–56 years old *N* (%)	*p*-value^a^
ACCEPTABILITY & USABILITY^c^	**Learnability**	Has anyone helped you to take the self-test?						<0.001
		* Yes*	30 (8.7%)	12 (27.9%)	18 (13.1%)	0 (0.00%)	0 (0.00%)	
		* No* ^b^	313 (91.3%)	31 (72.1%)	119 (86.9%)	124 (100%)	39 (100%)	
		Were you able to complete the self-test successfully?						1.000
		* Yes*	340 (99.7%)	43 (100%)	137 (99.3%)	121 (100%)	39 (100%)	
		* No* ^b^	1 (0.3%)	0 (0.00%)	1 (0.72%)	0 (0.00%)	0 (0.00%)	
		How easy or difficult did you find it to take the COVID-19 rapid antigen self-test?						0.075
		* Very easy + Slightly easy*	301 (87.8%)	35 (83.3%)	118 (84.3%)	109 (89.3%)	39 (100%)	
		* Neither easy nor difficult*	38 (11.1%)	7 (16.7%)	19 (13.6%)	12 (9.84%)	0 (0.00%)	
		* Slightly difficult + Very difficult*	4 (1.1%)	0 (0.00%)	3 (2.14%)	1 (0.82%)	0 (0.00%)	
		Reading success						0.215
		* Same outcome for participants* vs. *health workers*	270 (96.1%)	32 (94.1%)	85 (93.4%)	114 (97.4%)	39 (100%)	
		* Different outcome for participants* vs. *health workers*	11 (3.9%)	2 (5.88%)	6 (6.59%)	3 (2.56%)	0 (0.00%)	
	**Willingness**	How much do you agree or disagree with the following statement: “I would repeat the COVID-19 antigen rapid self-test in the future"?						0.136
		* Strongly agree + Slightly agree*	310 (94.2%)	34 (89.5%)	119 (91.5%)	120 (98.4%)	37 (94.9%)	
		* Neither agree nor disagree*	16 (4.86%)	3 (7.89%)	9 (6.92%)	2 (1.64%)	2 (5.13%)	
		* Slightly disagree + Strongly disagree*	2 (0.61%)	1 (2.63%)	1 (0.77%)	0 (0.00%)	0 (0.00%)	
		* Other*	1 (0.3%)	0 (0.00%)	1 (0.77%)	0 (0.00%)	0 (0.00%)	
		Would you be willing to repeat this self-antigen test twice a week if the epidemiological situation requires it?						0.008
		* Yes*	292 (94.5%)	29 (87.9%)	113 (91.1%)	113 (98.3%)	37 (100%)	
		* No*	17 (5.5%)	4 (12.1%)	11 (8.87%)	2 (1.74%)	0 (0.00%)	
		If you had to repeat the COVID-19 antigen test, you would prefer to do it in:						<0.001
		* Health care centre*	55 (17.7%)	14 (35.0%)	24 (18.6%)	13 (12.0%)	4 (11.8%)	
		* At home*	224 (72.0%)	22 (55.0%)	84 (65.1%)	88 (81.5%)	30 (88.2%)	
		* Others*	32 (10.3%)	4 (10.0%)	21 (16.3%)	7 (6.48%)	0 (0.00%)	
	**Suitability**	How much do you agree or disagree with the following statement: “I am confident that the reading of the result I have made is correct"?						0.030
		* Strongly agree + Slightly agree*	254 (92.4%)	29 (85.3%)	76 (89.4%)	114 (97.4%)	35 (89.7%)	
		* Neither agree nor disagree*	20 (7.3%)	5 (14.7%)	8 (9.41%)	3 (2.56%)	4 (10.3%)	
		* Slightly disagree + Strongly disagree*	1 (0.3%)	0 (0.00%)	1 (1.18%)	0 (0.00%)	0 (0.00%)	
		How much do you agree or disagree with the following statement: “I trust this type of self-test"?						0.465
		* Strongly agree + Slightly agree*	241 (86.7%)	31 (93.9%)	76 (84.4%)	102 (87.9%)	32 (82.1%)	
		* Neither agree nor disagree*	30 (10.8%)	2 (6.06%)	12 (13.3%)	12 (10.3%)	4 (10.3%)	
		* Slightly disagree + Strongly disagree*	7 (2.50%)	0 (0.00%)	2 (2.22%)	2 (1.72%)	3 (7.69%)	
	**Satisfaction**	What is your assessment of the experience of taking the COVID-19 antigen rapid self-test?						0.099
		* Very satisfied + Slightly satisfied*	307 (91.1%)	36 (90.0%)	119 (86.9%)	113 (93.4%)	39 (100%)	
		* Neither satisfied nor dissatisfied*	28 (8.31%)	4 (10.0%)	17 (12.4%)	7 (5.79%)	0 (0.00%)	
		* Slightly dissatisfied + Very dissatisfied*	2 (0.59%)	0 (0.00%)	1 (0.73%)	1 (0.83%)	0 (0.00%)	
		How much do you agree or disagree with the following statement: “I would recommend the COVID-19 rapid antigen self-test to a friend"?						0.774
		* Strongly agree + Slightly agree*	266 (94.0%)	33 (94.3%)	85 (93.4%)	112 (94.9%)	36 (92.3%)	
		* Neither agree nor disagree*	15 (5.3%)	2 (5.71%)	5 (5.49%)	6 (5.08%)	2 (5.13%)	
		* Slightly disagree + Strongly disagree*	2 (0.7%)	0 (0.00%)	1 (1.10%)	0 (0.00%)	1 (2.56%)	

^a^ *p*-value calculated using chi-square test.

^b^ Reasons for those who need help or did not succeed on sampling are included as a text in the results section of the manuscript.

^c^ The *Efficacy* subdomain is not included in the table because is defined as the ability to create a network with the main actors of the local health and education system -primary care, epidemiological surveillance service, educational community, local political agents-, correct communication of results, management of positive cases according to governmental protocols, and appropriate waste management, and results are presented in the aforementioned section.

A semi-structured interview was conducted with trained pupils who read the test results (School A) to assess the acceptability and usability of the experience, this was recorded and later transcribed for analysis.

### Data analysis

A descriptive analysis of the sample was carried out and the percentages of the categorical variables were calculated for each category, stratifying by age group in accordance with the educational stages of Catalonia based on Law 12/2009 of 10 July 2009 on education. The *p*-value was obtained by means of a Chi-square or Fisher test when the frequency was less than 5 using R software (version R-4.0.5).

### Ethics

Fundació Institut Universitari per a la recerca a l'Atenció Primària de Salut Jordi Gol i Gurina (IDIAPJGol) (code 20/192-PCV) and the Ethics and Clinical Research Committee of the Hospital Universitari Germans Trias (code PI-21-057), approved the study. Informed consent was obtained from all participants.

## Results

**Figure 1:**
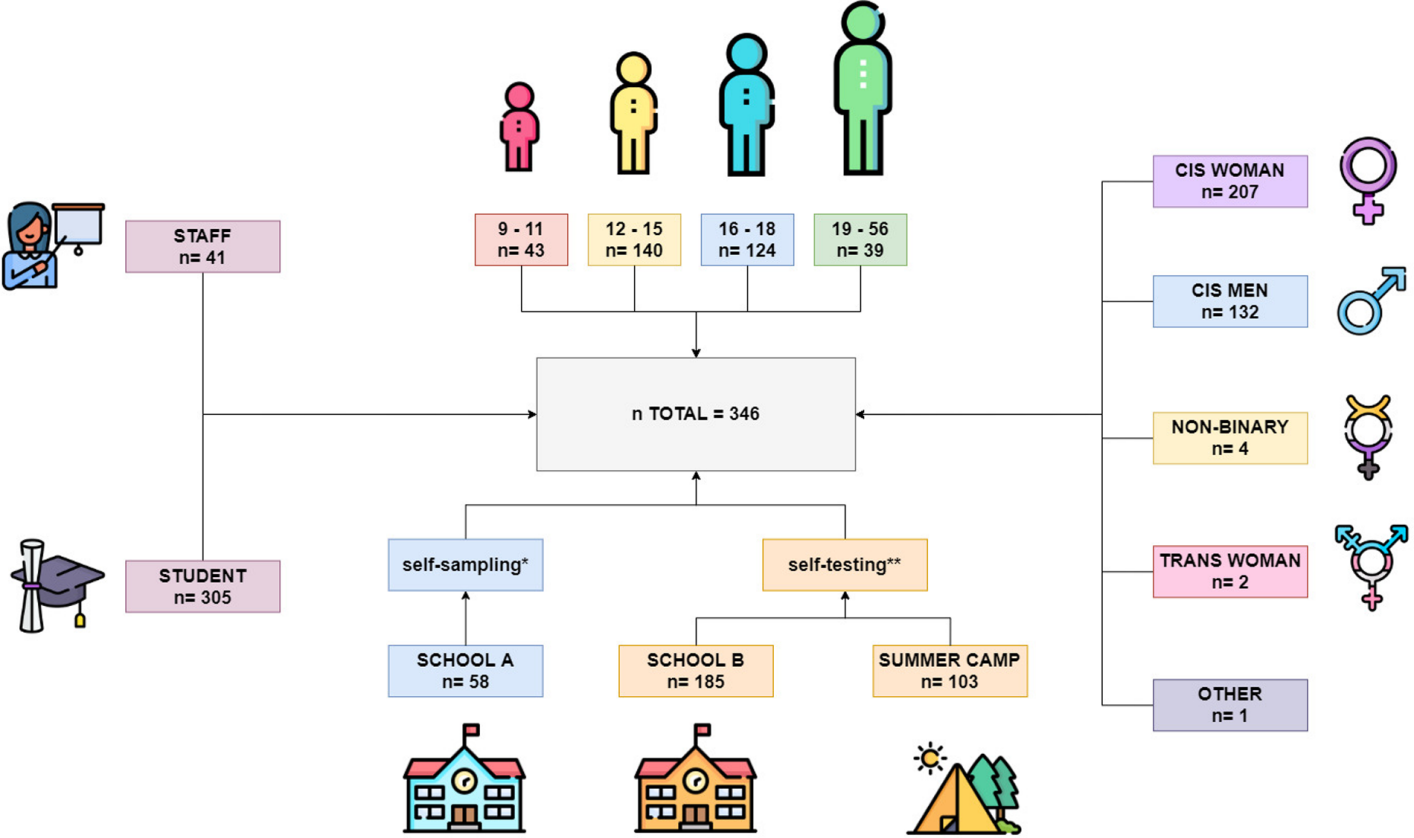
Summary of the total number of participants in the pilot study, Spain, N: 346. *Self-sampling: In school A, older pupils (15–16 years old) were trained beforehand to read the results of the other participants. ** Self-testing: In School B and summer camps, participants read the test result themselves, and the healthcare team validated the reliability.

A total of 305 students (88.15%) and 41 staff (11.85%) from 9 to 56 years old participated in the self-testing procedure and answered the survey at the end of the study. 207 were women (60%), 132 were men (38,3%), 4 non-binary people (1,16%), 2 transgender women (0,58%) and one participant with no information. Data was grouped in four age groups: 9–11 (*n* = 43; 12,4%), 12–15 (*n* = 140; 40,5%), 16–18 (*n* = 124; 35,8%), 19–56 (*n* = 39; 11,3%) (Figure 1).

Acceptability and usability subdomain results of the survey are presented in Table 1. Regarding the *Learnability* subdomain, 91.3% (*n* = 313) did not need help (Table 1), and those needing help mainly chose: *I was too nervous* (*n* = 14; 48,3%), *I needed help adding the buffer solution* (*n* = 2; 6,9%), *I needed help reading the result* (*n* = 2; 6,9%) or *Other reasons* (*n* = 9; 31%) (data not shown).

Regarding advantages, most participants identified: *Results within minutes* (*n* = 245; 86.0%), *Testing at school instead of at a health center* (*n* = 244; 85.6%) and *Tests improve safety and protection against Covid* (*n* = 224; 78.6%); followed by others such as *I can take control of my health with respect to Covid* (*n* = 191; 67.0%), *It gives me a more relaxed feeling when meeting friends* (*n* = 181; 63.5%), *Contributes to the normalization of Covid tests* (*n* = 164; 57.5%) and *The test is free of charge* (*n* = 160; 56.1%). Regarding disadvantages, the majority of participants identified that the test is *Less reliable than RT-PCR* (*n* = 213; 76.9%), while a few considered *You have to interpret the result yourself* (*n* = 48; 17.3%), or *Not having the emotional and/or logistical support to read the result* (*n* = 17; 6.14%) as disadvantages.

Regarding test reading, 96.1% of participants (*n* = 270) reported the same outcome as the healthcare workers (School B and summer camps). 11 participants (3.91%) scored a different outcome to the healthcare workers. Of these, 9 students (81,1%) indicated a negative result when the healthcare worker said positive, and 2 students (18,2%) answered that *they don't know the result,* when healthcare worker recorded a negative result.

In terms of *Efficacy*, there was a rapid response and proper management of positive cases, both at the educational and public health levels.

Through the semi-structured interview, the students of School A reported a *positive feeling about the research project*; that *the study helps to have a safer school* and *that a weekly test is acceptable*. However, trained students highlighted the heavy workload, with loss of class hours, and suggested involvement of more students in the future.

## Discussion

Overall, the study demonstrates high acceptability and usability of nasal swab Ag-RDT self-tests in students and staff in our settings. To our knowledge no previous results on feasibility in-depth account of acceptability and usability in younger groups in educational settings exist, although studies on specificity and sensitivity of this method do (8). Our results for adults (students and staff) agree with previous studies with rapid antigen and antibody tests (5, 9).

The results suggest that self-testing should be done under health workers or trained individuals' supervision in participants under 15 years of age; they needed more help and were less willing to repeat the test. Younger participants were also less confident about reading the result. In contrast to other studies, no statistical differences were found between age groups when reading the Ag-RDT result (10).

In addition, there was a high (more than 90%) agreement between self-reported and health care worker validated test readings.

The results also show that participants of all ages perceive the turnaround time and not having to travel to a health center as positive elements of the experience. It has been suggested that Ag-RDT self-testing can be a good tool for monitoring outbreaks, avoiding health care bottlenecks and improving access to diagnosis in places with less access to PCR laboratory tests. It also puts the patient at the center of the management of an infectious disease (3, 11). However, risk must be considered in the actions taken by each individual in the event of a positive case in relation to existing public health measures. Also, in an epidemic context, rapid tests are often developed and made commercially available at late stages, and therefore have to be considered as a medium to long term tool for outbreak management.

The study has some limitations to be noted. The number of participants was small -especially with regard to the representation of certain age groups- it is based on an opportunistic sample, and it would be interesting to include students of younger ages.

Due to the current massive use of Ag-RDT in different countries at the domestic level, more studies on feasibility of home-based testing in children, both self-testing and family testing are needed.

## Conclusions

The study demonstrates that SARS-CoV-2 antigen self-testing with nasal swabs is acceptable and usable for implementation in schools and summer camps with students and staff, when the epidemiological situation may require a systematic screening of these populations as demonstrated during the 5th Covid wave in Spain. These data have public health implications and remain of interest in case of emergence of new SARS-CoV-2 variants or other potential infectious agents. The strategy would relieve health centers work load, reducing the time it takes travel to health care centers for the educational community. However, in younger age groups supervision by healthcare or other previously trained in-school personnel is recommended.

## Data Availability

The raw data supporting the conclusions of this article will be made available by the authors, without undue reservation.
