# Corrigendum: Feasibility of severe acute respiratory syndrome coronavirus 2 (SARS-CoV-2) antigen self-testing in school and summer camp attendees

**DOI:** 10.3389/fped.2023.1353806

**Published:** 2024-01-05

**Authors:** Andreu Colom-Cadena, Hector Martínez-Riveros, Anna Bordas, Lucia Alonso-García, Marcos Montoro-Fernández, Pol Romano-deGea, Josep Vidal-Alaball, Elisabet Solà-Segura, Josep M. Llibre, Boris Revollo, Jordi Casabona, Cristina Agustí

**Affiliations:** ^1^ Centre of Epidemiological Studies on Sexually Transmitted Infections and AIDS of Catalonia (CEEISCAT), Ministry of Health, Government of Catalonia, Badalona, Spain; ^2^ Institut D’Investigació Germans Trias I Pujol (IGTP), Badalona, Spain; ^3^ Doctorate Program in Methodology of Biomedical Research and Public Health, Department of Pediatrics, Obstetrics and Gynecology and Preventive Medicine, Univ Autonòma de Barcelona, Badalona, Spain; ^4^ Health Promotion in Rural Areas Research Group, Gerència Territorial de la Catalunya Central, Institut Català de la Salut, Sant Fruitós del Bages, Spain; ^5^ Unitat de Suport a la Recerca de la Catalunya Central, Fundació Institut Universitari per a la Recerca a L'Atenció Primària de Salut Jordi Gol I Gurina, Sant Fruitós del Bages, Spain; ^6^ University of Vic-Central University of Catalonia, Vic, Spain; ^7^ Primary Heatlhcare Team Vic Nord, Institut Català de la Salut, Vic, Spain; ^8^ Division of Infectious Diseases and Foundation for Fighting AIDS, Infectious Diseases and Promoting Health and Science, University Hospital Germans Trias I Pujol, Badalona, Spain; ^9^ Departament de Pediatria, D’Obstetrícia I Ginecologia I de Medicina Preventiva I de Salut Publica, Universitat Autònoma de Barcelona, Bellaterra, Spain; ^10^ Spanish Consortium for Research on Epidemiology and Public Health (CIBERESP), Instituto de Salud Carlos III, Madrid, Spain

**Keywords:** acceptability and usability, SARS-CoV-2 antigen testing, SARS-CoV-2, school public health, self-testing

A Corrigendum on Feasibility of severe acute respiratory syndrome coronavirus 2 (SARS-CoV-2) antigen self-testing in school and summer camp attendees By Colom-Cadena A, Martínez-Riveros H, Bordas A, Alonso-García L, Montoro-Fernández M, Romano-deGea P, Vidal-Alaball J, Solà-Segura E, Llibre JM, Revollo B, Casabona J and Agustí C (2023). Front. Pediatr. 10:975454. doi: 10.3389/fped.2022.975454

Incorrect Affiliation

1. In the published article, there was an error in affiliation 3. Instead of “PhD in Methodology of Biomedical Research and Public Health, Department of Pediatrics, Obstetrics and Gynecology and Preventive Medicine, Univ Autonòma de Barcelona, Badalona, Spain”, it should be “Doctorate Program in Methodology of Biomedical Research and Public Health, Department of Pediatrics, Obstetrics and Gynecology and Preventive Medicine, Univ Autonòma de Barcelona, Badalona, Spain”.

